# In-vitro biofilm removal from TiUnite® implant surface with an air polishing and two different plasma devices

**DOI:** 10.1186/s12903-024-04230-9

**Published:** 2024-05-13

**Authors:** Sandra Haude, Rutger Matthes, Vinay Pitchika, Birte Holtfreter, Rabea Schlüter, Torsten Gerling, Thomas Kocher, Lukasz Jablonowski

**Affiliations:** 1 Department of Restorative Dentistry, Periodontology, Endodontology, Preventive Dentistry and Paediatric Dentistry, Dental School, University Medicine Greifswald, Walther-Rathenau-Str. 42a, Greifswald, D - 17475 Germany; 2 Imaging Center of the Department of Biology, University of Greifswald, Greifswald, Germany; 3 ZIK Plasmatis, Leibniz-Institute for Plasma Science and Technology e.V. (INP), a member of the Leibniz Research Alliance Leibniz Health Technology, Greifswald, Germany

**Keywords:** Air polishing, Biofilm, Cold plasma, Anodised titanium, Peri-implantitis, Surface treatment

## Abstract

**Background:**

We investigated the efficacy of two different cold atmospheric pressure jet plasma devices (CAP09 and CAPmed) and an air polishing device with glycine powder (AP) either applied as monotherapies or combined therapies (AP + CAP09; AP + CAPmed), in microbial biofilm removal from discs with anodised titanium surface.

**Methods:**

Discs covered with 7-day-old microbial biofilm were treated either with CAP09, CAPmed, AP, AP + CAP09 or AP + CAPmed and compared with negative and positive controls. Biofilm removal was assessed with flourescence and electron microscopy immediately after treatment and after 5 days of reincubation of the treated discs.

**Results:**

Treatment with CAP09 or CAPmed did not lead to an effective biofilm removal, whereas treatment with AP detached the complete biofilm, which however regrew to baseline magnitude after 5 days of reincubation. Both combination therapies (AP + CAP09 and AP + CAPmed) achieved a complete biofilm removal immediately after cleaning. However, biofilm regrew after 5 days on 50% of the discs treated with the combination therapy.

**Conclusion:**

AP treatment alone can remove gross biofilm immediately from anodised titanium surfaces. However, it did not impede regrowth after 5 days, because microorganisms were probably hidden in holes and troughs, from which they could regrow, and which were inaccessible to AP. The combination of AP and plasma treatment probably removed or inactivated microorganisms also from these hard to access spots. These results were independent of the choice of plasma device.

**Supplementary Information:**

The online version contains supplementary material available at 10.1186/s12903-024-04230-9.

## Background

The treatment of peri-implantitis is still a major problem and there are no generally accepted treatment guidelines. A recent Cochrane review did not find any debridement method superior to any other method in removing the biofilm [1] and no method was able to achieve clinically predictable, stable results over time [2–4]. The removal of biofilm from the exposed implant surface is regarded as the cornerstone of peri-implantitis therapy [1]. The exposed rough implant surface in combination with implant threads makes non-surgical treatment unpredictable [5, 6]. Today’s standard treatment for severe peri-implantitis is to expose the implant surface by a surgical flap and to remove the biofilm from the exposed implant surfaces [7]. Air powder devices showed the best cleansing capability of all mechanical methods. However, *in-vitro* studies have shown that up to 40% of the exposed surface remained untreated even during optimal access, especially in the undercuts of the implant threads [8–10]. The local use of antiseptic agents, air abrasives or lasers for decontamination of the implant surface during a surgical intervention did not improve the treatment outcomes compared with mechanical debridement combined with topical saline rinsing [11–13].

**Fig. 1:**
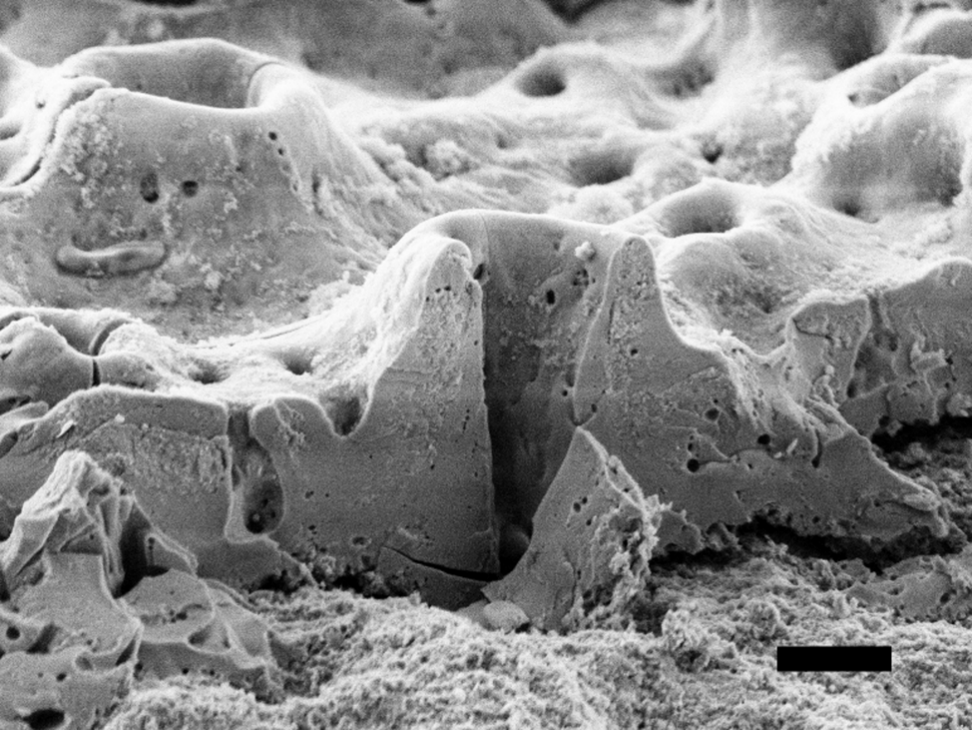
The TiUnite® surface is manufactured by spark anodisation in an electrolytic solution which produces an inner layer without pores and an outer layer with numerous pores with diameter and depth between ≤ 4 microns and ≤ 10 microns [15]. Black bar 2 μm

The rough implant surface and the implant threads provide ‘’protected areas’’ to the biofilm, inaccessible to conventional mechanical therapy. Therefore, surface decontamination is the critical step for the resolution of inflammation. Treatment of machined surfaces as originally devised by Brånemark displayed the best tendency for clinical healing, followed by sand-blasted, acid-etched surfaces, whereas TiUnite® surfaces showed less successful healing, which could be due to its unique surface characteristics (Fig. 1) [12, 14].

Physical plasma is formed when a gas is ionised. Plasma at atmospheric pressure is electrically neutral, composed of ions, electrons, vacuum ultraviolet and ultraviolet irradiation, free radicals, and chemically reactive neutral particles with a short lifespan and generates heat. Plasma inactivates planktonic bacteria in a dose-dependent anti-microbial effectivity [16, 17] and hydrophilises the exposed surface [18].

Plaque removal with cold jet plasma devices in combination with a brush or an air polishing device rendered sand-blasted, acid-etched titanium discs conducive for complete coverage with osteoblastic cells [19, 20]. Because of the complete coverage with cells, we appraised these treatment methods as successful. A new *in-vitro* study from our lab revealed that the topographically demanding anodised titanium surface used could not be treated as successfully as a sandblasted, acid-etched surface, used in a previous study [21].

Cold atmospheric pressure plasma devices (CAP) should not generate temperatures higher than 40 °C for treatment in or on patients [22, 23]. In our lab, we performed a series of studies with different plasma devices of the kINPen line, prototyped by the INP [24] (Leibniz-Institute for Plasma Science and Technology e.V., Greifswald, Germany). Our first experiments were performed with the kINPen08 [19], which generated too much heat but showed a successful biofilm removal in combination with a brush and was therefore replaced with the less powerful kINPen® 09 (neoplas GmbH, Greifswald, Germany). The plasma application of kINPen08 and kINPen® 09 did not consider medical regulations with respect to leakage current und temperature values. The kINPen® 09 was further modified to obtain CE approval for dermatological wound treatment (kINPen® MED, Class IIa CE certified, neoplas GmbH, Greifswald, Germany). Now we investigated if the medical compliant kINPen® MED [25] in combination with an air polishing device has an equal efficiency as the kINPen® 09 [20] and may overcome the topographical hurdles of a TiUnite® suface. In addition, physical differences of the plasma devices used in terms of radiation, temperature and thermal power were determined to understand possible different results between the two plasma sources kINPen® 09 and kINPen® MED and to make comparisons with the plasma source kINPen08 used in previous studies.

We hypothised that (i) there is no difference in biofilm removal and disinfection does not differ between the both CAP devices kINPen® MED and kINPen® 09 is used, and (ii) only the combined treatment with air polishing and CAP effectively removes the biofilm and disinfects the anodised titanium (TiUnite®) surfaces.

## Methods

### Characterisation of the cold atmospheric pressure plasma sources used

To characterise the difference between both plasma devices and to correlate with a previously used device iteration that showed positive biofilm reduction (kINPen08 [19]), optical plasma radiation, temperature and thermal power were determined in accordance with the DIN SPEC 91,315 [25]. The spectral irradiance was measured with a calibrated fibre optic spectrometer (AvaSpec-3648-USB2, Avantes, Apeldoorn, Netherlands), the fibre was combined with a cosine corrector to detect light from a bigger spatial range and placed end on in a distance of 10 mm from the capillary edge with a quartz plate shielding the fibre. For the temperature measurement a fibre optic temperature sensor (FOT Labor Kit, LumaSense Technologies, Inc. GmbH, Santa Clara, USA) was placed at different positions in front of the devices. Furthermore, the fibre optic temperature sensor was surrounded by a light cupper plate to determine the time dependent heating of the plate. By calorimetric evaluation, the thermal power of the plasma was determined at different positions in front of the devices.

### Titanium discs

Titanium discs with an anodised TiUnite® surface (5 mm diameter and 1 mm thickness) were used (Nobel Biocare AB, Göteborg, Sweden). The lateral production-related holes were filled with a light-curing composite (Venus Pearl A2, Kulzer, Hanau, Germany) and polymerised under a light-curing device for 10 min (Biodent VLC440, Dentsply, York, United States). Subsequently, after cleaning for 15 min in an ultrasonic bath in distilled water (S 30 H elmasonic, Elma Schmidbauer GmbH, Singen, Germany), the discs were sterilised (Tuttnauer 2540 EK, Breda, Netherlands) and dried for 20 min.

### Biofilms cultivation

The subgingival plaque was harvested with curettes from deep pockets of a volunteer (male, periodontally diseased), placed into a tube with culture media (Schaedler Boulion, Carl Roth, Karlsruhe, Germany) and incubated for 24 h at 37 °C / 5% CO_2_ to serve as inoculum for biofilm. The institutional review board of University Medicine Greifswald has approved plaque removal (registration number: BB 094/19). After 24 h, the titanium discs were placed into 96-well microtitre plates (Techno Plastic Products AG, Trasadingen, Switzerland), covered with 100 µl of the precultured, subgingival plaque solution and were cultivated for 7 days in an incubator (37 °C, 5% CO_2_). Every day medium was renewed, and the discs were dried for 20 min under lamina flow in an airflow cabinet. After biofilm culture, the biofilm-covered discs were transferred into new wells of a sterile microtitre plate for treatment.

### Experimental setup

Seven test groups were assessed, the five treatment groups air polishing (AP), cold atmospheric pressure plasma with the kINPen® 09 (CAP09), kINPen® MED (CAPmed), combination of air polishing and kINPen® 09 (AP + CAP09), or kINPen® MED (AP + CAPmed), and two control groups, the sterilised and untreated discs without prior biofilm cultivation as positive control (PC), and discs with untreated biofilm as negative control (NC). The treatments were repeated in 5 runs with 4 discs each (*n* = 20) for each of the 7 groups (Σ = 140). All 20 discs of one group were treated at day 0, and *n* = 10 per group were evaluated at day 0 by fluorescence microscopy. The other 10 discs were placed in a culture medium and were evaluated after 5 days of cultivation at 37 °C / 5% CO_2_ (day 5) by fluorescence microscopy. Scanning electron microscopical evaluation was additionally performed on day 0 and day 5 on discs of test run 4.

### Air polishing treatment (AP)

We used a powder-water air polishing device (AIRFLOW Master Piezon®, EMS, Nyon, Switzerland) with glycine powder (particle size 25 mm, connected to the dental unit (air pressure 4.75 bar, water pressure 2.5 bar). The device was run at full water pressure and quarter power. The handpiece was fixed in a holder and hovered at a vertical distance of 5 mm +/- 1 mm under an angle of 80° over the disc. Each side of a disc was first treated at 4 equidistantly distributed spots for 10 s and then in a meandering movement for 20 s, thus complete treatment time amounted to 60 s per side. After treatment, the disc was rinsed with 2 ml of 0.9% sodium chloride solution and placed in new sterile microtitre plate for further experimental steps.

### Cold atmospheric pressure plasma treatment (CAP)

The titanium discs were treated with two different jet plasma sources (kINPen® 09, neoplas GmbH, or kINPen® MED, neoplas MED GmbH, Greifswald, Germany). Both plasma devices were running at a frequency of approx. 1 MHz [24]. They were comparable in principle, except that the kINPen® MED operated with a duty cycle of 50% (on/off) and a repetition frequency of 2.5 kHz [24]. The carrier gas used was argon (99.999%, ALPHAGAZ, Air Liquide, Düsseldorf, Germany). The flow regulator (MKS Instruments, Munich, Germany) controlled the gas flow of 5 slm (standard litres per minute). For both devices, the length of the visible plasma plume was set to 10 mm with a circular effective area on the surface > 10 mm in diameter with an intensity profile dropping exponentially from the center to the edge [25]. The devices were gradually moved to 9 spots in a small circular motion by a computer-controlled table. The distance between the disc surface and the nozzle of the pen was 5 mm indicating the conductive mode operation [26]. One spot was in the middle and the other eight in the periphery. Each spot was treated for 60 s, amounting to 540 s of one side. The discs were treated on both sides to prevent re-growth of microbes from the back side.

### Biofilm regrowth

The treated and the untreated control titanium discs for the evaluation after day 5 were placed in 96-well microtitre plates (Techno Plastic Products AG, Trasadingen, Switzerland) covered with 100 µl sterile biofilm culture medium and cultured for 5 days in the incubator (37 °C, 5% CO_2_). The medium was replaced daily, and the discs were dried for 20 min in an airflow cabinet. After 5 days, the biofilm regrowth was evaluated.

### Fluorescence evaluation of residual biofilm

The treated and the untreated control titanium discs were analysed with fluorescence microscopy (Olympus BX60, 2x magnification, GFP filter, Olympus U-RFL-T, Hamburg, Germany) by digital images taken with a camera (SLR; EOS 450D, Canon, Krefeld, Germany, Program: M, Tv: 0.5 s, ISO: 200/24°, WB: Manually, jpg: L (large)). To assess residual biofilm the discs were stained with 10 µM SYTO™ 9 Green Fluorescent Nucleic Acid Stain (Thermo Fisher Scientific, Eugene, Oregon, USA) for 30 min at room temperature in dark. Thereafter, the dye was removed, and the discs were washed with 300 µl distilled water and then dried before microscopy. The images were evaluated with the software ImageJ (v1.50, US National Institutes of Health, Bethesda, MD, USA). The area (region of interest), mean gray value and integrated density were measured to. Therefore, the specimen parameters mean area, and integrated density were all measured, and the data were transferred to a spreadsheet program.

### Scanning electron microscopy (SEM)

After the fluorescent evaluation, one disc per each test group of run 4 was submitted to the sample preparation for scanning electron microscopy. Samples were fixed (2.5% glutaraldehyde in PBS) and then treated with 2% tannic acid in washing buffer (100 mM cacodylate buffer [pH 7.4], 1 mM calcium chloride, 25 mM sodium azide) for 1 h, 1% osmium tetroxide in washing buffer for 1 h and 1% thiocarbohydrazide for 30 min at room temperature - with washing steps in between. After treatment with 1% osmium tetroxide in washing buffer overnight at 4 °C, the samples were dehydrated in a graded series of aqueous ethanol solutions (10%, 30%, 50%, 70%, 90%, 100%) on ice for 15 min each step. The samples were then allowed to reach room temperature before the ethanol was replaced with fresh 100% ethanol at room temperature for 10 min. Subsequently, samples were critical point-dried with liquid CO_2_. Finally, samples were mounted on aluminium stubs, sputtered with gold/palladium and examined with a scanning electron microscope EVO LS10 (Carl Zeiss Microscopy GmbH, Oberkochen, Germany). All micrographs were edited by using Adobe Photoshop CS6.

For evaluation, a 20x overview image was taken of each disc, as well as a representative image with 2,000x magnification of the disc according to Matthes et al. [20].

### Statistical analyses

To calculate the relative biofilm fluorescence factor score (BfF), firstly, the mean background value was calculated for the PC group (positive sterile control) individually for day 0 and day 5 (day 0 = 21.67 ± 1.66, day 5 = 13.94 ± 1.35), assuming that differences in mean scores between both assessment days should not influence the results. Following this, the BfF was calculated for all test discs using the following formula: “Integrated Density – (Area of selected specimen X mean background value of the respective PC)”. The values are dimensionsless numbers based on the gray tone histogram of the images.

Distributional differences in the relative BfF between days (Table 1) and groups (Supplementary Table 1) were tested using Mann-Whitney U tests. P-values were adjusted for multiple testing by Benjamini Hochberg false discovery rate method [27]. Furthermore, to assess the effects of different treatment protocols and their interactions on the relative BfF factors, linear regression models stratified by day were constructed, using relative BfF factors in million grayscale units as the dependent variable regressing over AP, CAP and the interaction between AP and CAP. Because the run was significantly associated with the BfF, it was included as a covariate. Predicted relative BfF factors for all combinations of AP and CAP devices were graphically shown (estimates with 95% confidence intervals).

**Table 1:** Distribution of relative biofilm fluorescence factor values [in million grey scale values] on titanium discs treated with different methods and assessed immediately (Day 0) and after culturing the treated discs in the medium for 5 more days (Day 5)

Treatment	Day 0	Day 5	p-value
PC	0.89 (-7.40; 5.95) (-15.63-21.01)	-4.70 (-5.01; 8.58) (-13.08-13.71)	0.870
NC	952.32 (895.69; 1043.60) (671.89–1105.00)	172.80 (98.82; 225.19) (50.56-442.63)	**< 0.001**
CAP09	704.17 (687.58; 804.75) (631.13–847.50)	534.99 (499.98; 627.57) (427.20-739.06)	**0.002**
CAPmed	672.04 (612.64; 777.49) (561.97-846.98)	640.58 (509.64; 679.75) (228.72-709.87)	0.096
AP	-51.12 (-58.93; -30.07) (-65.48-74.15)	444.93 (344.26; 533.17) (293.63-614.25)	**< 0.001**
AP + CAP09	-21.68 (-42.22; -5.53) (-59.00-42.28)	-10.70 (-16.74; 227.44) (-23.41-457.43)	**0.036**
AP + CAPmed	-40.86 (-47.29; -23.91) (-53.58-37.41)	-8.34 (-13.96; 263.41) (-29.79-609.62)	**0.003**

Data are presented as median (25%; 75% quantiles) and (5-95% quantiles). PC: Positive control (sterile discs), NC: Negative control (biofilm covered discs), CAP09: cold atmospheric pressure plasma treatment using kINPen® 09, CAPmed: cold atmospheric pressure plasma treatment using kINPen® MED; AP: air polishing. Pairwise comparisons between Day 0 and Day 5 were made using Mann-Whitney-U test. Bold numbers indicate statistically significant difference

P-values < 0.05 in bold were considered statistically significant. All statistical analyses were performed using Stata/SE 14.2 [28] and R version 4.2.2 [29].

## Results

### Initial comparison of physical parameters between CAP09 and CAPmed

**Fig. 2:**
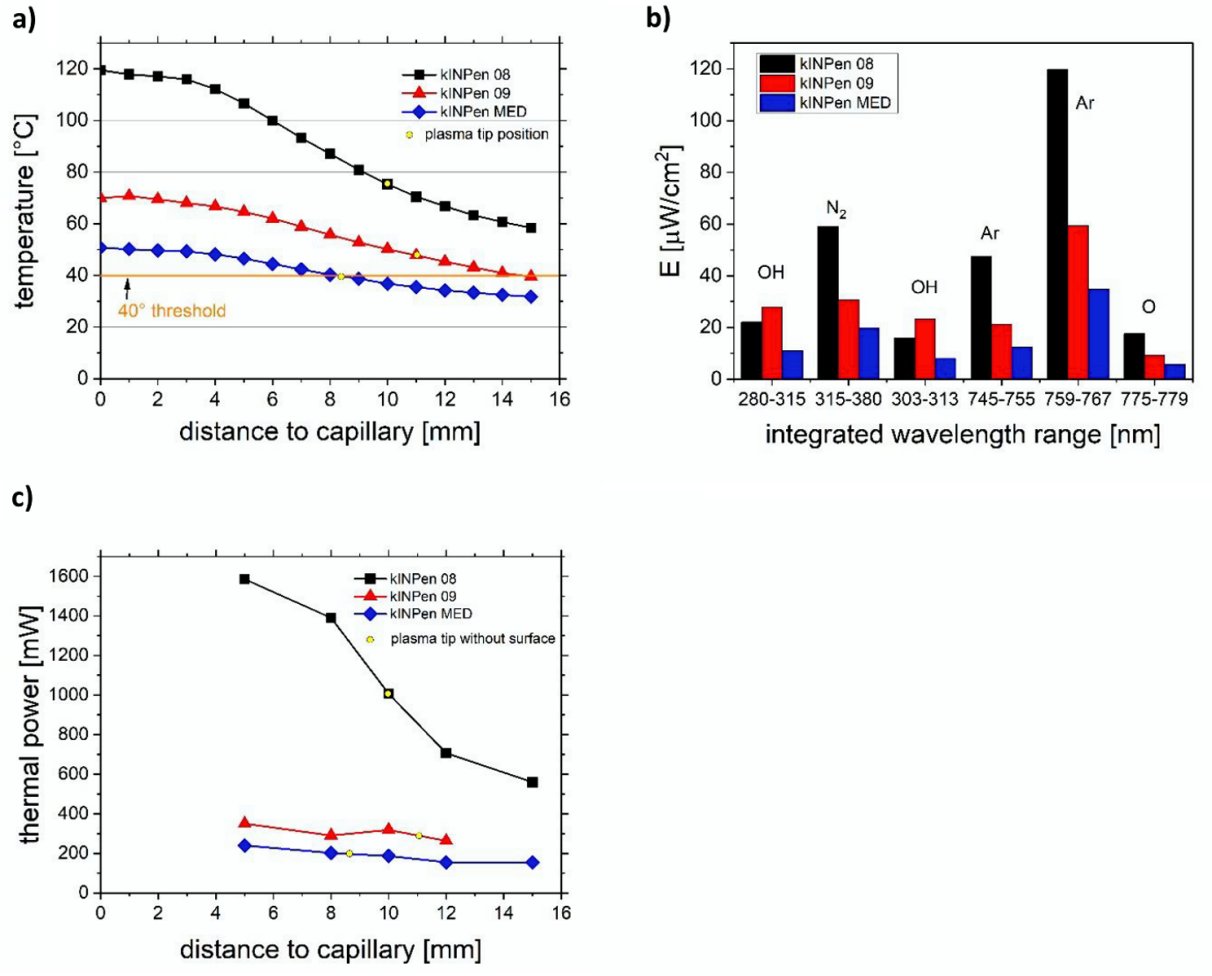
Showing basic physical device parameters to compare the investigated kINPen® 09 and kINPen® MED with each other and the previously used kINPen08. (**a**) temperature profile over the distance to the capillary; (**b**) emissivity for individual spectral ranges; (**c**) thermal power on a treated surface over distance

The measured physical parameters of the CAP09 and CAPmed devices of the generated temperature, emission spectrum and thermal power as a function of distance to the capillary, and additionally for comparison with the kINPen08 device used in previous studies, are shown on Fig. 2. The thermal power of up to 1.6 W for the kINPen08 however was effective in terms of biofilm removal. Now comparing these values with the CAP09 and CAPmed in the present study, both devices had significantly lower temperature and thermal power values, ranging from 70 °C (CAP09) and 50 °C (CAPmed) at the nozzle and 45 °C (CAP09) and 40 °C (CAPmed) at the effluent tip. The thermal power was below 0.3 W for CAP09 and around 0.2 W for CAPmed. Based on thermal perspective alone, the old kINPen08 version generated a power 2- and 4-fold higher compared to CAP09, while CAPmed`s power was about two thirds of CAP09. This difference in power output was previously investigated for this discharge geometry to depend on the operation frequency, here with a shift from 1 MHz for the CAP09 and CAPmed, while the kINPen08 is operated at 2 MHz [24, 30]. The emission is used as a relative tracer for reactive species like reactive oxygen and nitrogen species. Based on the measurements, all lines except for the hydroxyl radical showed a similar tendency as the thermal power, with kINPen08 generating two-fold the signal for molecular nitrogen, atomic argon and atomic oxygen compared to CAP09, while CAPmed emission was about two thirds of CAP09.

### Results of biofilm removal on Day 0 and biofilm regrowth on Day 5

**Fig. 3:**
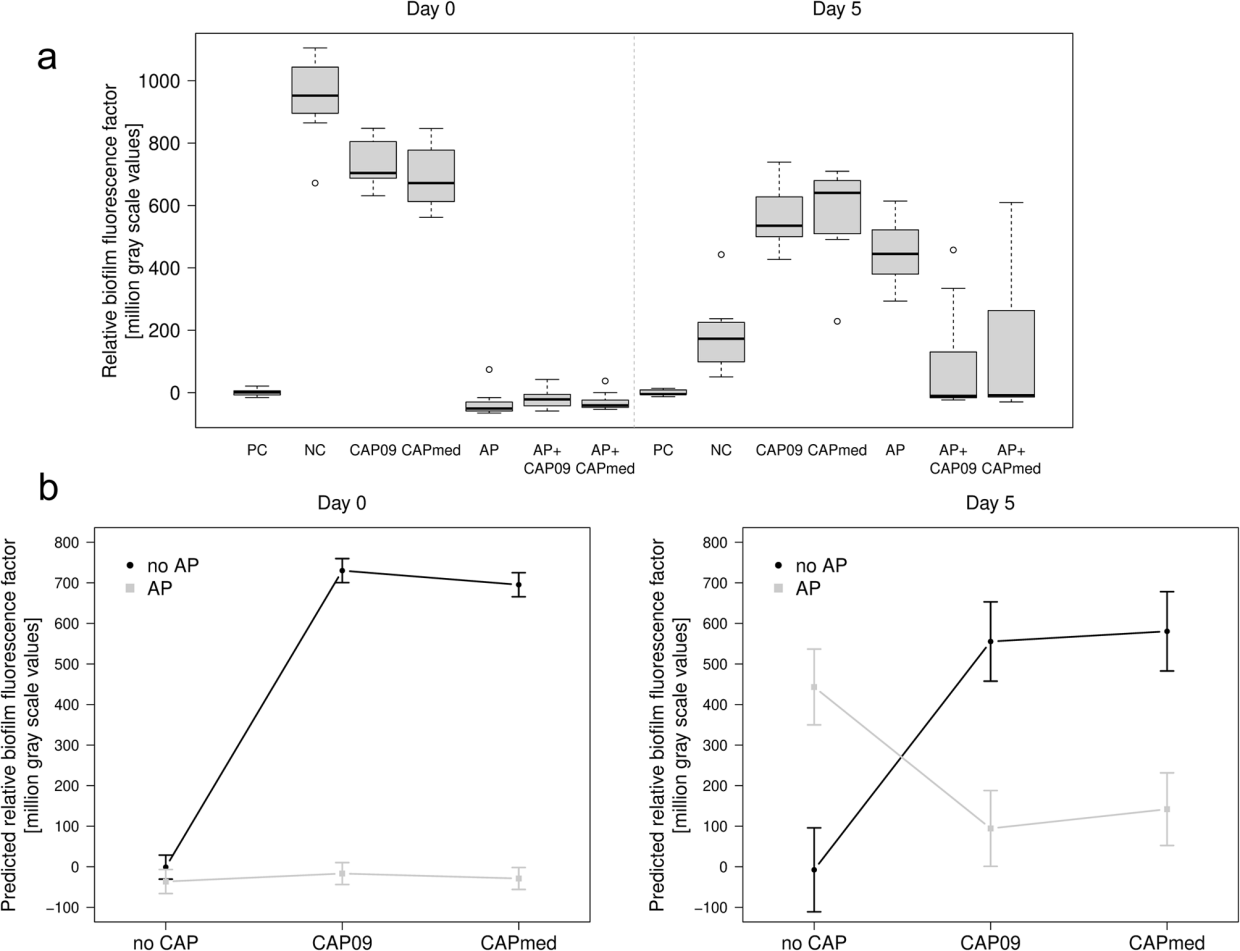
(**a**) Boxplot for relative biofilm fluorescence factors by treatment groups on day 0 and day 5. Treatment groups: positive control (PC; sterile discs), negative control (NC; biofilm covered discs), cold atmospheric pressure plasma treatment using kINPen® 09 (CAP09), cold atmospheric pressure plasma treatment using kINPen® MED (CAPmed); air polishing (AP); combined treatment of AP + CAP with kINPen® 09 AP + CAP09 and the combined treatment of AP + CAP with kINPen® MED (AP + CAPmed). Fluorescence signals identify microbial residues. (**b**) Relative biofilm fluorescence factors predicted from linear regression models according to air polishing (AP) and pressure plasma treatment (CAP) on days 0 and 5. Fluorescence signals identify microbial residues. Abbreviations: CAP09, cold atmospheric pressure plasma treatment using kINPen® 09, CAPmed, cold atmospheric pressure plasma treatment using kINPen® MED; AP, air polishing

On day 0 the median BfF scores of all treatment groups were significantly different from the NC (untreated biofilm) (952.32) and PC (sterile discs) (0.89) (Table 1; Fig. 3a, Supplementary Table 1). The BfF value of the NC (952.32) was only marginally higher than the BfF values of CAP09 (704.17) and CAPmed (672.04). The BfF values of AP (-51.12), AP + CAP09 (-21.68), AP + CAPmed (-40.86) were even lower than for the PC (0.89; *p* < 0.05), reflecting the presumptive absence of biofilm on these disc. Despite a higherspreading of BfF scores being wider in the combination treatment groups (AP + CAP09 and AP + CAPmed), their median BfF values remained below 0 for both groups AP + CAP09 (-21.7) and AP + CAPmed (-40.9), and was smaller compared to PC (0.9; *p* < 0.05).

Comparing the BfF values of Day 0 and Day 5 (Table 1; Fig. 3, Supplementary Table 1), distributions did not differ significantly for PC (0.89 vs. -4.70) and CAPmed (640.6 versus 672.04), while for NCs (952.32 vs. 172.80) and CAP09 (534.99 versus 704.17), significant differences were detected in combination with higher BfF values at day 0. For AP (444.93 versus − 51.12), AP + CAP09 (-10.70 versus − 21.68) and AP + CAPmed (-8.34 versus − 40.86), BfF values differed significantly between day 0 and day 5, but with higher values observed at day 5.

Results from mixed linear regression models confirmed (Table 2) revealed that effects on BfF values were most pronounced for combinations of AP + CAP09 and very close of AP + CAPmed (Fig. 3b). The interaction displays lower β values (-711.31, -688.67) compared tp the AP alone (-35.50) regarding the sterile PC (Table 2).

**Table 2:** Results from linear regression models evaluating the effects of different treatments on biofilm fluorescence factor reported in million grey scale values

Variable	Day 0		Day 5	
	β (95% CI)	p-value	β (95% CI)	p-value
AP (Ref.: no AP)	-	-	-	-
AP	-35.50 (-77.39; 6.39)	0.095	450.76 (311.48; 590.04)	< 0.001
CAP treatment (Ref.: no CAP treatment)	-	-	-	-
CAP09	731.00 (689.10; 772.89)	< 0.001	562.80 (420.51; 705.10)	< 0.001
CAPmed	696.19 (654.30; 738.08)	< 0.001	587.94 (445.64; 730.23)	< 0.001
Interaction between AP and CAP treatment	-	-	-	-
AP x CAP09	-711.31 (-769.35; -653.26)	< 0.001	-911.61 (-1105.50; -717.72)	< 0.001
AP x CAPmed	-688.67 (-746.71; -630.62)	< 0.001	-889.20 (-1081.40; -697.01)	< 0.001
run (Ref.: 1)	-	-	-	-
2	-70.47 (-107.39; -33.54)	< 0.001	-113.46 (-237.39; 10.46)	0.072
3	-83.95 (-122.19; -45.71)	< 0.001	-152.41 (-274.55; -30.28)	0.015
4	-58.98 (-95.90; -22.05)	0.002	-50.78 (-174.70; 73.14)	0.415
5	-38.48 (-76.72; -0.23)	0.049	-113.24 (-234.89; 8.42)	0.067

*Abbreviations* β, regression coefficient; CI, confidence interval; AP: air polishing; CAP09: cold atmospheric pressure plasma treatment using kINPen® 09, CAPmed: cold atmospheric pressure plasma treatment using kINPen® MED

**Fig. 4:**
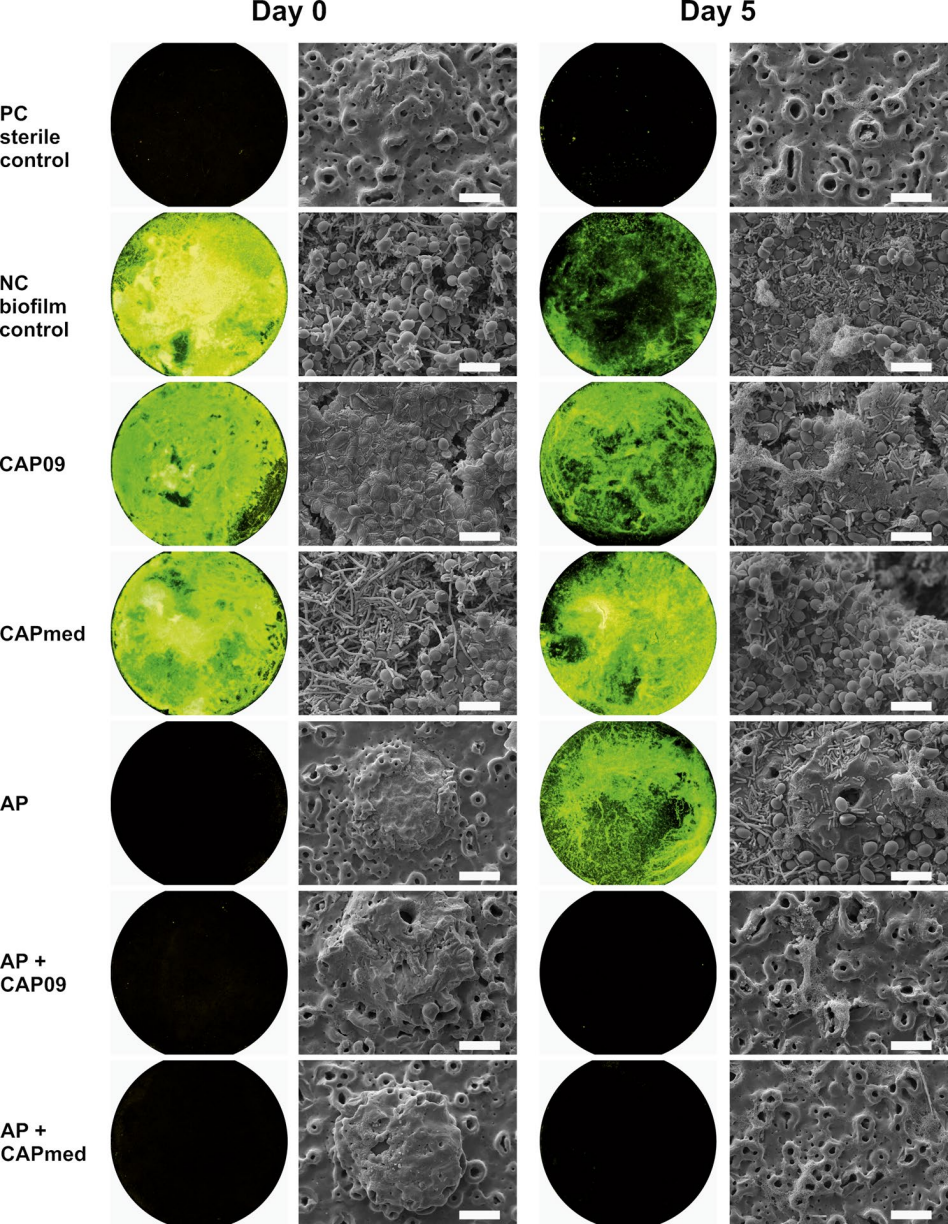
Fluorescence microscopy images (left; 2x magnification) and scanning electron micrographs (right; 2,000x magnification) directly after treatment (Day 0) and after 5 days of cultivation (Day 5) for the test groups. PC: Positive control (sterilised and untreated discs without prior biofilm cultivation); NC: Negative control (discs with untreated biofilm); CAP09: cold atmospheric pressure plasma treatment using kINPen® 09; CAPmed: cold atmospheric pressure plasma treatment using kINPen® MED; AP: air polishing; AP + CAP09 and AP + CAPmed: combined treatment of AP + CAP with kINPen® 09 and kINPen® MED, respectively. Fluorescence signals show microbial residues. Scale bars = 10 μm

On Day 5, estimates were higher in all standalone treatment groups (AP: 450.8; CAP09: 562.8; CAPmed: 587.9) than the positive control. In line with the results from Day 0, estimates were significantly lower than the stand-alone treatments CAP, AP), when AP and either form of CAP treatment was combined (AP x CAP09: -911.6; AP x CAPmed: -889.2) (Table 2). Furthermore, on both days, the runs were significantly affecting the estimates. Fluorescence microscopy images for each treatment group for Days 0 and 5 are presented in Fig. 4.

### Scanning electron micrographs

The sterilised and untreated discs (PC) did not show any biofilm on Day 0 and Day 5 (Fig. 4), and the TiUnite® surface with its characteristic pores surrounded by a volcano-shaped uplift was clearly visible. The pores had a diameter of 2 to 4 μm (Fig. 1). On the NC disc a solid microbial biofilm could be detected on Day 0. On Day 5, the biofilm was reduced and did not cover the total surface. Mono treatment with CAP09 and CAPmed showed a crusty, indistinctive agglomerate of microorganisms. The orginal topography was hidden under a microbial layer embedded in a matrix. The AP treated discs did not show any microorganisms on Day 0, but on Day 5 a sparse biofilm covered the surface. Discs treated with AP + CAP09 or AP + CAPmed had comparable surface characteristics to the sterile control surface on both Day 0 and Day 5 (kinen4). Distinct oval walled holes protruded volcano-like from the outer layer. Taken together, on Day 0 good cleaning results were visible with AP and AP + CAP instrumentations. However, on Day 5 only the combined treatment modalities with AP + CAP09 and AP + CAPmed displayed a surface almost without microorganisms.

## Discussion

In our previous investigation the kINPen08 was able to reduce microbial biofilm [19], but the 2 MHz operation frequency of the kINPen08 generated temperatures outside the device of up to 120 °C and around 85 °C in the tip region (Fig. 2). Therefore, this device was not suitable for medical applications, and we focused here on the well investigated plasma devices kINPen® 09 and kINPen® MED. In this study, the combined instrumentation of AP along with either CAP09 or CAPmed prevented microbial regrowth on Day 5 on 74% of the TiUnite® discs, with BfF values equal to the pristine control discs (values were even or lower), whereas on the other 26% of the discs microbial regrowth was observed (Fig. 3; Table 1). Although the initial AP treatment on Day 0 resulted in BfF values as low as these on the pristine control discs and the scanning electron microscopy corroborated this positive outcome, mono AP treatment could not prevent regrowth on all discs on Day 5. While AP does not reach into the holes, a gas-driven plasma device can penetrate the holes with diffusion of e.g. long-living reactive oxygen and nitrogen species as observed with OES. These are generated by both devices, kINPen® 09 and kINPen® MED with only minor changes in intensity (Fig. 2b).

Probably single bacteria were hidden in the pores and holes, from which they overgrew the disc again during the 5-day period. This observation is in line with a recent study from our lab, where we treated acid-etched and TiUnite® surfaces with a combination of AP + CAP (kINPen08). On all sand-blasted, acid-etched surfaces no biofilm was detected after a 5-day regrowth period (BfF values were comparatively low as on control discs), whereas on most TiUnite® discs regrowth occurred [21]. Taken together these results indicate, that the instrumentation of a TiUnite® surface is much more demanding than the one of a sand-blasted, acid-etched surface. In line with former publications from our lab, mono CAP treatment, irrespective of CAP modifications (kINPen08, kINPen® 09, kINPen® MED) did not result in any treatment success [20, 31]. As well in line with a recent clinical study, a sole mechanical treatment with air polishing did not yield successful results [4]. The results show that negative control was strongly reduced at Day 5 compared to Day 0 samples. This effect was not observed in previous studies, so we do not have a clear explanation at this time. Perhaps the biofilm cycle entered a dispersion state associated with loosening and facilitated removal of the biofilm matrix [32]. Because of the greatly reduced biofilm mass of Day 5 NC, it is not fully comparable to other Day 0 test groups. In contrast, the microorganisms remaining after treatment of the surfaces will be in the proliferation stage, so we assume that there is full comparability with other test groups and time points here. Supplementary it should be mentioned that the low values for the NC at Day 5 leads to reduced differences to other test groups, which increases the requirements for the determination of statistical differences, which in turn increases the significance for the test groups of Day 5.

The TiUnite® surface is characterised by a thick, porous outer TiO_2_ layer enriched with highly crystalline calcium phosphate, which is osteoconductive and promotes and speeds up osseointegration [33]. These pores were up to 10 μm deep [34]. This advantage of rapid and advanced bone formation during the healing phase comes along with the drawback, that a TiUnite® surface hampers cleaning. While the implant surface characteristics seems to have no influence on initiation of peri-implantitis [35], some dog and human studies indicate, that the progression of peri-implantitis was greater in implants with TiUnite® surface than with acid-etched sufaces [36, 37]. We suspect, residual bacteria were hidden in these pores after AP treatment, which regrew between Day 0 and Day 5. Our data present an explanation for the observation in dog studies [36, 38], why mechanical treatment of TiUnite® surfaces did not result in resolution of the inflammation, and why bone loss progressed despite treatment, whereas at mechanically treated turned or acid-etched surfaces inflammation was resolved and progression of bone loss stalled. These animal studies were confirmed in a 3-year clinical study, which showed that surgical periimplantitis treatment of TiUnite® surface resulted in less probing depth reduction than that of turned or acid-etched surfaces and TiUnite® implants had a higher risk for progression of peri-implantitis [12, 39]. To overcome this problem a radical treatment approach was investigated in a dog study: the removal of the outer layer with a bur and with citric acid eradicated the bacteria in these hideaways and was superior to the use of photodynamic therapy, Er: Yag laser or only bur [40]. These data point towards a greater demand for development of a more efficient therapy of implants with a TiUnite® surface than for sand-blasted, acid-etched surfaces.

The fact that the negative control group (biofilm control) had a smaller BfF after a further 5 days of cultivation than after the first 7 days of cultivation did not meet our expectations. On the one hand, this could be due to the fact that the regrown biofilm was washed off during the daily medium change or fluorescence staining procedure or was inhibited in their growth for an unknown reason. We did not observe this in our similar previous experiments.

A conference proceeding in 2016 summarised that air polishing with glycine powder could disrupt biofilms from implants without any surface damage [41, 42]. These air polishing features raised the hope in the dental community to have a debridement procedure at hand, which may overcome the inherent problems of implant instrumentation. A 12-month retrospective study reported a very moderate 8% success irrespective of debridement with air polishing or cotton pellet soaked with saline [43]. A subsequent RCT of the same group with a 6 month follow up compared three different debridement protocols during surgery (plastic curette, titanium brush, air polishing with glycine powder); only 29% of treated implants with air polishing, 22% plastic curette, 33% titanium brush were rated as successful [44]. A similar disappointing success rate of 33% was reported in 12-month follow-up RCT [4]. These low success rates are in agreement with a success rate of 34% in implants with modified surfaces [45].

A recent review, based on 11 studies, summarised the present knowledge about the effects of *in-vitro* CAP treatment of microbially contaminated implants [46], with additional three studies published in the meantime. Three studies reported that monotherapy with CAP did not confer any beneficial aspect [18, 31, 47] and that additional use of AP was necessary to completely remove biofilm, whereas two studies did not find any additional effect of CAP besides AP application [20, 48]. To what extent other different CAP constructions or CAP settings (power, gas feeding, gas sort, distance) are more effective than our devices, is open to debate. Other electrochemical methods with a similar mechanism of action are also currently being investigated in vitro [49, 50]. Currently new methods are under investigation in laboratory and pre-clinical application that could be efficient methods to achieve higher success rate in open flap peri-implantitis therapy, the electrolytical based [51, 52] and the water stream combined with cold atmospheric plasma based implant cleaning procedures [53].

Our experimental procedure needs to be discussed. In the literature, there is a multitude of *in-vitro* studies, which examined the removal of bacteria directly after instrumentation [50, 54–57]. As we have shown in our lab, inspection or assessment of residual microorganisms directly after treatment (corresponding to Day 0 in our experiment) gives biased answers, because microorganisms are hidden in the cavities and need time to regrow [58]. We suggest observing either microbial regrowth after 5 days after debridement or alternatively to seed osteoblasts on the debrided surface to examine if cells can cover the surface or were overgrown by microorganisms. We assume this microbial regrowth as one of the major reasons for insufficient healing. Conclusions drawn from these *in-vitro* studies without follow up observation period lull us into a false sense of security.

Our biofilm model is often criticised. We acknowledge, that this model only marginally reflects an *in-vivo* situation, but we do not want to shed light on biofilm composition or microbial colonisation on implants. The only purpose was to have a layer of microbial multi-species biofilm firmly attached to a titanium surface, which could not be washed off with running tap water, and with which we could check different instrumentation procedures. We regard our model much closer to any artificial plaque model, in which a lacquer or nail polish simulates plaque [9].

## Conclusions

This study examined the effectiveness of two different CAP devices (kINPen® 09 and kINPen® MED) with and without prior AP treatment on biofilm covered TiUnite® surfaces. Our hypothesis that the combined treatment with AP and CAP leads to a complete removal of the biofilm from TiUnite® surfaces from all discs was shown directly after treatment. However, it was not confirmed after long term observation, because very clean but no sterile surface could be achieved. Furthermore, there was no difference in cleansing or antimicrobial effectivity between the two CAP devices despite CAPmed providing only two thirds thermal power and radiation intensity compared to CAP09.

### Electronic supplementary material

Below is the link to the electronic supplementary material.

**Supplementary Table 1**: Pairwise comparison of different methods at Day 0 and Day 5 using Mann-Whitney-U test.

## Data Availability

The datasets used and/or analysed during the current study are available from the corresponding author on reasonable request. The datasets generated and/or analysed for regulatory approval, which were not explicitly discussed in this study, are partially not publicly available due international regulations.

## Abbreviations

AP: powder-water air polishing
BfF: biofilm fluorescence factor score
CAP: cold atmospheric pressure plasma
CAP09: cold atmospheric pressure plasma with the plasma source kINPen® 09
CAPmed: cold atmospheric pressure plasma with the plasma source kINPen® MED
NC: negative control
PC: positive control
SEM: Scanning electron microscopy
