# Supplementary material for: In-vitro biofilm removal from TiUnite® implant surface with an air polishing and two different plasma devices
Source: BMC Oral Health. 2024 May 13;24:558. doi: 10.1186/s12903-024-04230-9 (PMC11089677; doi:10.1186/s12903-024-04230-9)
Supplement: Supplementary file 1 — Supplementary Table 1: Pairwise comparison of different methods at Day 0 and Day 5 using Mann-Whitney-U test. [file 12903_2024_4230_MOESM1_ESM.docx]

**Supplementary Table 1.** Pairwise comparison of different methods at Day 0 and Day 5 using Mann-Whitney-U test

| **Test method** | **Comparative method** | **Day 0** | | **Day 5** | |
| --- | --- | --- | --- | --- | --- |
|  |  | **p-value** | **BH adjusted**  **p-value** | **p-value** | **BH adjusted**  **p-value** |
| CAP09 | Sterile  Positive control | 0.0002 | **0.0005** | 0.0002 | **0.001** |
| CAPmed |  | 0.0002 | **0.0004** | 0.0002 | **0.001** |
| AP |  | 0.0025 | **0.0034** | 0.0002 | **0.002** |
| AP+CAP09 |  | 0.0409 | **0.0486** | 0.7903 | 0.790 |
| AP+CAPmed |  | 0.0030 | **0.0038** | 0.3556 | 0.397 |
| CAP09 | Biofilm Negative control | 0.0015 | **0.0022** | 0.0003 | **0.001** |
| CAPmed |  | 0.0007 | **0.0011** | 0.0003 | **0.001** |
| AP |  | 0.0002 | **0.0004** | 0.0004 | **0.001** |
| AP+CAP09 |  | 0.0001 | **0.0010** | 0.0486 | 0.058 |
| AP+CAPmed |  | 0.0001 | **0.0006** | 0.0479 | 0.058 |
| CAP09 | AP | 0.0002 | **0.0003** | 0.0411 | 0.056 |
| CAPmed |  | 0.0002 | **0.0003** | 0.0167 | **0.026** |
| AP+CAP09 |  | 0.0649 | 0.0725 | 0.0004 | **0.001** |
| AP+CAPmed |  | 0.1469 | 0.1551 | 0.0193 | **0.028** |
| CAP09 | CAPmed | 0.3258 | 0.3258 | 0.4057 | 0.428 |
| AP+CAP09 |  | 0.0001 | **0.0005** | 0.0002 | **0.001** |
| AP+CAPmed |  | 0.0001 | **0.0004** | 0.0012 | **0.002** |
| AP+CAP09 | CAP09 | 0.0001 | **0.0003** | 0.0002 | **0.001** |
| AP+CAPmed |  | 0.0001 | **0.0003** | 0.0056 | **0.010** |

BH: Benjamini-Hochberg; CAP09: cold atmospheric pressure plasma treatment using KINPen® 09, CAPmed: cold atmospheric pressure plasma treatment using KINPen® MED; AP: air polishing. Pairwise comparisons between different treatment methods in each day were made using Mann-Whitney-U test. Bold numbers indicate statistically significant differences after adjusting for multiple testing.
